# Protein microarray analysis identifies key cytokines associated with malignant middle cerebral artery infarction

**DOI:** 10.1002/brb3.746

**Published:** 2017-07-07

**Authors:** Zhonghe Zhou, Jinghua Zhang, Xiaoqiu Li, Cheng Xia, Yaling Han, Huisheng Chen

**Affiliations:** ^1^ Department of Neurology General Hospital of Shen‐Yang Military Region Shenyang China; ^2^ Department of Cardiology General Hospital of Shen‐Yang Military Region Shenyang China

**Keywords:** cytokines, functional enrichment analysis, Malignant middle cerebral artery infarction, microarray, protein–protein interaction

## Abstract

**Introduction:**

We aimed to explore potential cytokines involved in the malignant middle cerebral artery infarction (MMI) and elucidate their underlying regulatory mechanisms.

**Methods:**

We first developed a cytokine profile by Quantibody^®^ Human Cytokine Antibody Array7000 using serum samples from eight patients with MMI and eight patients with non‐acute cerebral infarction (NACI). The differentially expressed cytokines were then identified in patients with MMI using two‐tailed Student's *t*‐test and Fisher's Exact Test compared with patients with NACI. Gene Ontology and pathway enrichment analyses were performed using DAVID. Protein–protein interaction (PPI) network was constructed based on STRING database.

**Results:**

A total of 10 differentially expressed cytokines were identified from 320 unique inflammatory cytokines in serums. Among them, four cytokines, like NCAM1 (neural cell adhesion molecule 1), IGFBP‐6 (insulin‐like growth factor binding protein 6), LYVE1 (lymphatic vessel endothelial hyaluronan receptor 1), and LCN2 (Lipocalin2), were up‐regulated, while another six cytokines, such as TGFB1 (transforming growth factor, beta 1, also known as LAP), EGF (epidermal growth factor), PDGFA (platelet‐derived growth factor alpha polypeptide), MMP‐10 (matrix metallopeptidase 10), IL‐27 (interleukin 27), and CCL2 (chemokine (C‐C motif) receptor 2), were down‐regulated. Moreover, cytokine–cytokine receptor interaction pathway was significantly enriched.

**Conclusions:**

Our findings indicate that 10 differentially expressed cytokines, such as NCAM1, LCN2, IGFBP‐6, LYVE1, MMP‐10, IL‐27, PDGFA, EGF, CCL2, and TGFB1 may participate in the development of MMI. Moreover, cytokine–cytokine receptor interaction pathway may be an important mechanism involved in this disease. These differentially expressed cytokines may serve as diagnostic biomarkers or drug targets for MMI.

## 1 INTRODUCTION

Malignant middle cerebral artery infarction (MMI), a common acute cerebral infarction, is the most devastating form of ischemic stroke with an 80% mortality rate (Huttner and Schwab 2009; Vahedi et al., 2007). It is characterized with large supratentorial infarcts caused by acute occlusion of malignant middle cerebral artery and space‐occupying brain edema followed by cerebral herniation (Berrouschot, Sterker, Bettin, K02ster, & Schneider, 1998; Hacke, Schwab, Horn, Spranger, & De GM, 1996). Patients with cerebral infarction caused by either thrombi or emboli always suffer irreversible neurologic deficits which markedly lower the quality of daily living (Kim et al., 2000). In addition, multiple risk factors can contribute to cerebral infarction, such as high blood pressure, diabetes, and tobacco smoking (Hankey, 2006). However, the pathogenesis of this disease has not been fully elucidated to date.

In recent years, many approaches, such as thrombolytic drugs, mechanical embolectomy, and angioplasty have been introduced in the therapy of acute cerebral infarction, however, exploration of effective treatments for MMI remains a great challenge in neurocritical care (Huttner and Schwab 2009). A previous work has suggested that early craniectomy may be more effective since a reduction in mortality from 27% to 16% can be caused by stepwise reduction in time from 39 to 21 hours between surgery and stroke onset (Schwab, 1998). Moreover, decompressive surgery (hemicraniectomy) has been reported to effectively improve neurological outcome and decrease the mortality in patients with MMI when it is performed within 48 hr of stroke onset (Minnerup et al., 2011). Therefore, identification of key mechanisms involved in MMI will be of great significance for the early diagnosis and treatment of MMI.

Ischemic injury of brain tissues and subsequent necrosis and apoptosis of nerve cells are shown to be the main reason for cerebral infarction (Frizzell, 2005; Mergenthaler, Dirnagl, & Meisel, 2004). Cranial computerized tomography (CT) findings as major early signs also show that ischemia covering is more than 30% at occlusion site (Sparacia, Iaia, Assadi, & Lagalla, 2007). Recently, accumulated evidences have stressed the important roles of inflammatory reaction mediated by cytokines in the pathophysiology of acute brain ischemia (Sotgiu et al., 2006; Vila, Castillo, Dávalos, & Chamorro, 2000). The plasma levels of some inflammatory cytokines, such as tumor necrosis factor (TNF‐α) and interleukin (IL)‐6, are reported to be a predictive diagnosis of acute ischemic stroke in the acute setting (Tuttolomondo, Di Raimondo, di Sciacca, Pinto, & Licata, 2008). In addition, cytokines are up‐regulated in the brain after stroke and can recruit more circulating leukocytes for enlarging the cerebral infarct area though infiltrating the ischemic region and promoting the loss of neuronal cells and brain tissue (Feuerstein, Wang, & Barone, 1998). Various cytokines are demonstrated to be involved in the neurogenesis and/or neuronal regeneration (Dirnagl, Simon, & Hallenbeck, 2003; Schäbitz et al., 2003). Besides, administration of hematopoietic cytokines, like stem cell factor and granulocyte colony‐stimulating factor in the subacute phase after cerebral infarction is shown to be effective for enhancing cytokine‐induced generation of neuronal cells and may serve as a new therapeutic strategy (Kawada et al., 2006). Although considerable advances have been made, key cytokines associated with MMI development have not been definitely determined, as well as their regulatory mechanism is still largely unknown.

In this study, we developed a protein microarray to detect the concentrations of 320 unique inflammatory cytokines simultaneously in serum of patients with MMI and patients with non‐acute cerebral infarction (NACI). Then the differentially expressed cytokines in serum of patients with MMI were identified. Besides, functional enrichment analyses and protein–protein interaction (PPI) network analysis were performed. The objective of our study was to explore potential cytokines involved in the MMI and to elucidate their possible regulatory mechanisms in MMI development.

## 2 MATERIALS AND METHODS

### 2.1 Patients

A total of eight patients with MMI and eight patients with NACI were recruited. MMI was diagnosed according to the following criteria: signs on CT showed an infarct of at least 50% of the middle cerebral artery territory or infarct volume >145 cm³ on diffusion‐weighted images (DWIs); secondary neurological deterioration including consciousness decline was defined by at least 1 point of consciousness item described in the National Institutes of Health Stroke Scale (NIHSS), which was an essential parameter to assess the severity of acute ischemic stroke; patients were hospitalized within 24 hr of disease onset; patients had no history of other brain disorders, such as hemorrhagic cerebral vessel disease, idiopathic epilepsy, intracranial infection, or craniocerebral trauma; and patients had no other infections such as urinary tract infections or pneumonia. Meanwhile, NACI was defined as non‐acute cerebral infarction (7 to 14 days after onset) and had no any clinical sequelae after 6 months. The infarct volume of NACI patients was confirmed according to T2‐weighted magnetic resonance imaging. In addition, the clinical baseline data of the above two different types of diseased population was shown as follows: the median age was 68 ± 3.36 (range: 57–80) vs. 70.75 ± 2.51 (range: 61–79) years, the ratio of male/female was all 1:1. The risk factors of patients are shown in Table 1. The baseline NIHSS of MMI group and NACI group was 24.375 ± 1.89 and 4.0 ± 1.12, and infarct volume by DWI in above two groups was 154.75 ± 7.11 and 12.38 ± 3.69, respectively.

**Table 1:** The risk factors of the participants

Risk factors	Patients with MMI (*N *= 8)	Patients with NACI (*N *= 8)
Diabetes	3	2
Coronary heart disease	4	3
Hypertension	5	6
Artery atherosclerosis	6	6
Stroke	4	2
Smoking	4	4
Drinking	4	4

The blood collection of the included MMI patients was performed at 24 hr of stroke onset. The blood samples of NACI patients were collected at the next morning after admission. The serum collection from these patients was performed as perviously described. In brief, 10 milliliters of peripheral blood sample was collected from median cubital vein of each individual and then centrifuged at 1000 *g* for 15 min. Finally, the serum fraction was separated, aliquotted, and stored at −80°C for further use. Our study was approved by Ethics Committee of General Hospital of Shenyang Military Area Command and written informed consent was obtained from each patient or their responsible families.

### 2.2 Protein microarray design and analysis

An antibody‐based cytokine array system (Quantibody^®^ Human Cytokine Antibody Array 7000, RayBiotech, Inc., Norcross, GA, USA) was used to detect multiple cytokines simultaneously in serum of patients with MMI (*n *= 8) and patients with NACI (*n *= 8). This array system utilized the multiplexed sandwich ELISA‐based technology to quantitatively measure the concentration of 320 human cytokines. Briefly, an equal amount of sample diluent was added into each well and incubated for 30 min to block slides. Then multiple cytokine‐specific capture antibodies together with the positive controls were arrayed onto the glass support in quadruplicate. After incubation for 2 h with gentle shaking, the diluted serum samples were incubated with the second biotinlabeled specific detection antibody to recognize a different isotope of the target cytokine. The streptavidin‐labeled Cy3 equivalent dye was then added to incubate in dark room for 1 h, and the signals were subsequently visualized using a laser scanner. Data extraction was done using microarray analysis software GenePix laser scanner (Toronto, Canada), and Quantibody^®^ Q‐Analyzer software (RayBiotech, Inc., Norcross, GA, USA) was used for quantitative data analysis. By comparing signals from positive controls, the cytokine concentration in each serum was finally determined based on the standard curves generated by 8‐point cytokine concentrations.

### 2.3 Analysis of differential protein expression

Analysis of continuous variables and categorical variables between these cytokines were, respectively, carried out using the two‐tailed Student's *t*‐test or Fisher's Exact Test (χ^*2*^) test with SPSS version19.0 (SPSS Inc., Chicago, IL, USA) . The differentially expressed cytokines in serum of patients with MMI were then identified compared with patients with NACI. A value of *p *< .05 was considered statistically significant. Moreover, the fold change in the individual cytokine expression in serum of patients with MMI compared to that in serum of patients with NACI was calculated to distinguish up‐ and down‐regulation of cytokines. Fold change >1 represented that this cytokine was up‐regulated in serum of patients with MMI compared with patients with NACI, while fold change <1 represented down‐regulation.

### 2.4 Functional enrichment analyses

Gene Ontology (GO, http://www.geneontology.org) (Ashburner et al., 2000) is widely used for unification of biology of a large scale of genes and proteins. Kyoto Encyclopedia of Genes and Genomes (KEGG, http://www.genome.ad.jp/kegg/) (Kanehisa & Goto, 2000) database is widely used for correlating gene sets into their respective pathways. Reactome (http://www.reactome.org) (Croft et al., 2014) is a curated, peer‐reviewed resource of human biological pathways and reaction network. The presumed inherited direction (PID) (Schaefer et al., 2009), a pathway interaction database, mainly focus on signaling pathways. BioCarta (http://www.biocarta.com) (Nishimura, 2001) is an interactive online resource, which is divided into three main categories: gene function, proteomic pathway, and reagent exchange. PANTHER pathway (Mi & Thomas, 2009) is an ontology‐based pathway database for illustrating how ontologies and standards play a role in data analysis via coupling with data analysis tools.

In our study, to analyze these differentially expressed cytokines in functional level, we performed GO annotation, KEGG pathway, Reactome, PID, BioCarta, and PANTHER pathway enrichment analyses using Database for Annotation, Visualization, and Integrated Discovery (DAVID, http://david.abcc.ncifcrf.gov/) (Dirnagl et al., 2003), which was an online tool for functional annotation and visualization of large‐scale genomic or transcriptomic data. A *p*‐value <.05 was set as the threshold value.

### 2.5 PPI network construction

Search Tool for the Retrieval of Interacting Genes (STRING) (Szklarczyk et al., 2011) is an online database providing comprehensive information of predicted and experimental interactions of proteins. The PPI pairs in STRING database are displayed with a combined score. In our study, these differentially expressed cytokines were mapped into STRING database with combined score >0.5 for identifying the significant PPI pairs. PPI network was then established and visualized using Cytoscape software (Kohl, Wiese, & Warscheid, 2011). Degree centrality (Jeong, Mason, Barabási, & Oltvai, 2001) as a topological property of PPI network was then used to identify hub nodes in the network. The higher the degree was, the more important the nodes were in the network.

## 3 RESULTS

### 3.1 Cytokine profiling

In our study, Quantibody^®^ Human Cytokine Antibody Array7000 was used to simultaneously measure the concentrations of 320 unique inflammatory cytokines in serum of patients with MMI and patients with NACI. Data extraction was done using microarray analysis software and the cytokine profiling was developed for subsequent analysis.

### 3.2 Identification of differentially expressed cytokines

Based on the expression value of each cytokine, 10 differentially expressed cytokines were identified in serum of patients with MMI compared with patients with NACI. Among them, four cytokines, including NCAM1 (neural cell adhesion molecule 1), IGFBP‐6 (insulin‐like growth factor binding protein 6), LCN2 (Lipocalin2), and LYVE1 (lymphatic vessel endothelial hyaluronan receptor 1) were up‐regulated, while another six cytokines, like TGFB1 (transforming growth factor, beta 1, also known as LAP), MMP‐10 (matrix metallopeptidase 10), IL‐27 (interleukin 27), EGF (epidermal growth factor), PDGFA (platelet‐derived growth factor alpha polypeptide), and CCL2 (chemokine (C‐C motif) receptor 2, also known as MCP‐1), were down‐regulated.

### 3.3 Functional enrichment analyses

We performed GO enrichment analysis for functional annotation of these differentially expressed cytokines. As shown in Table 2, these differentially expressed cytokines were significantly enriched in GO functions related with hyaluronan metabolic process (3.90E‐05), regulation of hyaluronan biosynthetic process (7.96E‐05), platelet alpha granule lumen (9.48E‐05), platelet alpha granule (1.95E‐04), and secretory granule lumen (1.95E‐04). The top 10 GO terms are shown in Table 2.

**Table 2:** The top 10 Gene Ontology terms enriched by differentially expressed cytokines

Category	Term	Count	*p*‐value
GO:0030212	Hyaluronan metabolic process	3	3.90E‐05
GO:1900125	Regulation of hyaluronan biosynthetic process	2	7.96E‐05
GO:0031093	Platelet alpha granule lumen	3	9.48E‐05
GO:0031091	Platelet alpha granule	3	1.95E‐04
GO:0034774	Secretory granule lumen	3	2.04E‐04
GO:0030213	Hyaluronan biosynthetic process	2	2.57E‐04
GO:0030214	Hyaluronan catabolic process	2	3.38E‐04
GO:0031983	Vesicle lumen	3	3.74E‐04
GO:0060205	Cytoplasmic membrane‐bounded vesicle lumen	3	3.74E‐04
GO:0002576	Platelet degranulation	3	4.93E‐04

In addition, the significantly enriched KEGG, Reactome and PID pathways of these differentially expressed cytokines were shown in Table 3. The results showed that the significantly enriched KEGG pathways were cytokine–cytokine receptor interaction, malaria, and glioma. The significantly enriched Reactome pathways were extracellular matrix organization, nonintegrin membrane‐ECM interactions and ECM proteoglycans. The markedly enriched PID pathways were IL27‐mediated signaling events, ceramide signaling pathway and PDGF receptor signaling network. However, BioCarta and PANTHER pathways were not significantly enriched.

**Table 3:** The top 10 enriched pathways by differentially expressed cytokines via different databases

Category	Term	Count	*p*‐value
KEGG pathways			
hsa04060	Cytokine–cytokine receptor interaction	4	.0100043
hsa05144	Malaria	2	.0110871
hsa05214	Glioma	2	.0186208
hsa05212	Pancreatic cancer	2	.0191468
hsa05218	Melanoma	2	.0218693
hsa04540	Gap junction	2	.0328786
hsa05215	Prostate cancer	2	.0328786
hsa05323	Rheumatoid arthritis	2	.0342118
hsa05142	Chagas disease (American trypanosomiasis)	2	.0433727
hsa04010	MAPK signaling pathway	3	.0477863
Reactome			
REACT_118779	Extracellular matrix organization	7	2.56E‐07
REACT_163874	Non‐integrin membrane‐ECM interactions	7	3.77E‐07
REACT_163906	ECM proteoglycans	9	1.77E‐05
REACT_318	Platelet degranulation	6	4.38E‐04
REACT_6323	Host Interactions with Influenza Factors	4	4.06E‐03
REACT_1280	Response to elevated platelet cytosolic Ca2+	11	2.37E‐06
REACT_120996	Hyaluronan uptake and degradation	11	4.70E‐05
REACT_120726	TGF‐beta receptor signaling in EMT (epithelial to mesenchymal transition)	6	7.15E‐04
REACT_121083	Hyaluronan metabolism	4	3.05E‐03
REACT_115852	Signaling by constitutively active EGFR	6	4.77E‐03
PID			
il27pathway	IL27‐mediated signaling events	2	.0051456
ceramide_pathway	Ceramide signaling pathway	2	.0134937
pdgf_pathway	PDGF receptor signaling network	1	.0267852
telomerasepathway	Regulation of Telomerase	2	.0289511
ap1_pathway	AP‐1 transcription factor network	2	.0313181
et_egfrpathway	EGFR‐dependent Endothelin signaling events	1	.0380509
erbb_network_pathway	ErbB receptor signaling network	1	.0602047
syndecan_1_pathway	Syndecan‐1‐mediated signaling events	1	.0674790
rxr_vdr_pathway	RXR and RAR heterodimerization with other nuclear receptor	1	.0995446
alk1pathway	ALK1 signaling events	1	.0995446

**Figure 1:**
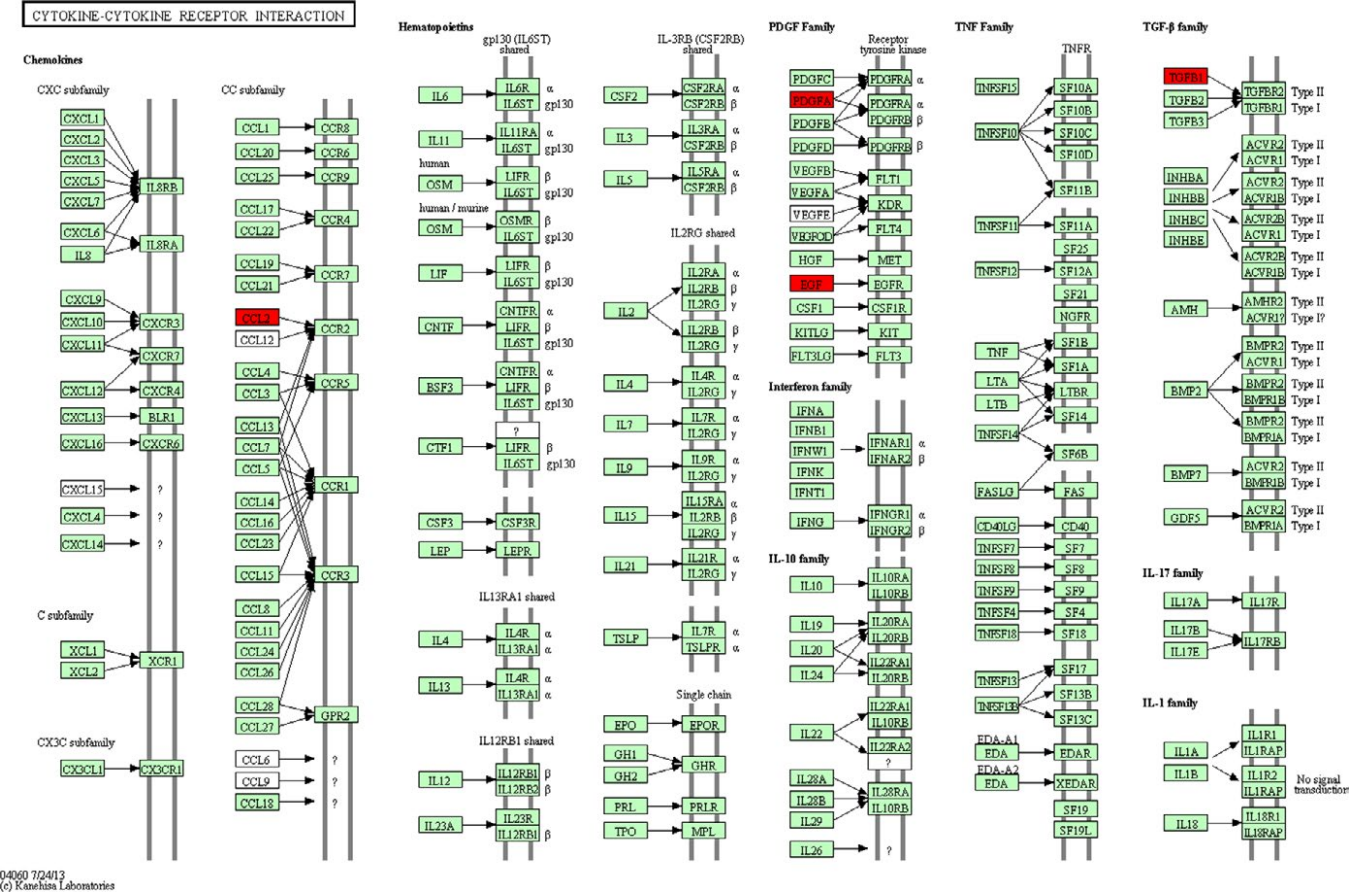
Cytokine–cytokine receptor interaction pathway. The red nodes are the screened differentially expressed cytokines in our study

Besides, the detailed pathway information of cytokine–cytokine receptor interaction is shown in Figure 1, which displayed the reaction network of several cytokine families, such as chemokines, PDGF family, and TGF‐β family. We found that the important differentially expressed cytokines in chemokines was CCL2, in PDGF family were PDGFA and EGF, and in TGF‐β family was TGFB1. Notably, these cytokines were all down‐regulated.

### 3.4 PPI network analysis

**Figure 2:**
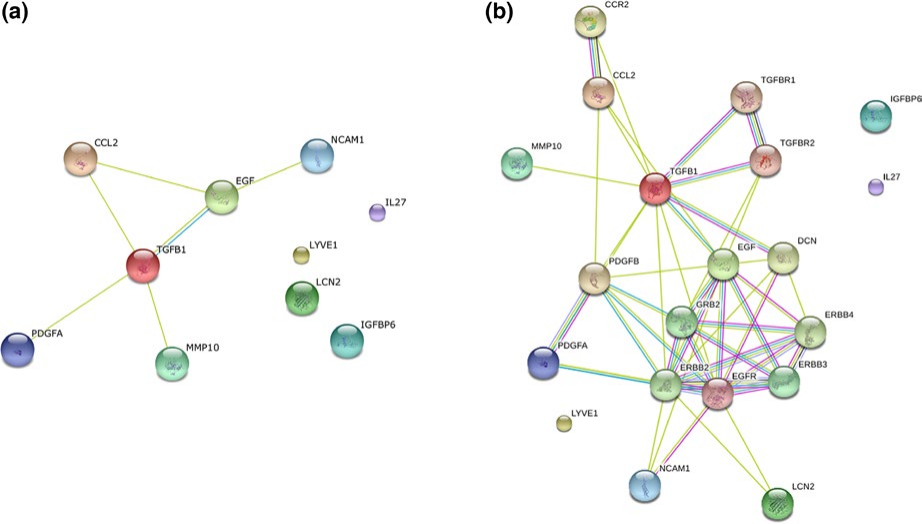
Protein –protein interaction (PPI) networks. (a) PPI network was constructed by the screened differentially expressed cytokines in our study; (b) PPI network was constructed by differentially expressed cytokines and other interacted proteins

Based on the information of STRING database, the PPI network constructed by the above 10 differentially expressed cytokines was obtained (Figure 2a). The results showed that TGFB1 (degree = 4) could interact with the most cytokines, followed by EGR (degree = 3) and CCL2 (degree = 2). Furthermore, we identified another 10 nodes based on the combined score of PPI pairs >0.5, which could interact with the above 10 differentially expressed cytokines directly. Then total 20 nodes were used to construct another PPI network. As shown in Figure 2b, EGF, EGFR (epidermal growth factor receptor), GRB2 (growth factor receptor‐bound protein 2), ERBB2 (v‐erb‐b2 erythroblastic leukemia viral oncogene homolog 2), and ERBB3 (v‐erb‐b2 erythroblastic leukemia viral oncogene homolog 3) were identified as hub nodes in this PPI network.

## 4 DISCUSSION

Microarrays analysis has been shown to measure the concentrations of many proteins simultaneously, which dramatically accelerates the pace of discovery of drug targets and biomarkers (Wilson & Nock, 2003). In this study, we developed cytokine profile using peripheral blood of MMI and NACI patients and microarray data were analyzed to detect the concentrations of 320s cytokines simultaneously. The results showed that 10 differentially expressed cytokines in serum of patients with MMI compared with patients with NACI were identified. Among them, NCAM1, IGFBP‐6, LCN2, and LYVE1 were up‐regulated, while TGFB1, MMP‐10, IL‐27, EGF, PDGFA, and CCL2 were down‐regulated. Moreover, down‐regulated cytokine, such as PDGFA, EGF, CCL2, and TGFB1 were significantly enriched in cytokine–cytokine receptor interaction. The crucial roles of these cytokines in the pathogenesis of MMI and their potential values in the diagnosis and treatment of this disease merit further discussion.

Increasing evidence has proved that cytokines usually act as inflammatory mediators involved in ischemic cascade and result in an inflammatory state (Tuttolomondo et al., 2008). Cytokines are also involved in various disease processes, including MMI (Chu et al., 2014). In our study, the concentrations of NCAM1, LCN2, IGFBP6, and LYVE1 were significantly higher in patients with MMI compared with those in patients with NACI. NCAM1 as a member of the immunoglobulin superfamily is reported to be implicated in the expansion of T cells and dendritic cells which play a role in immune surveillance, and is identified as key genes associated with ischemic stroke (Wang et al., 2013). Tur et al. also demonstrated that up‐regulation of the 140‐kD isoform of CD56 (NCAM1) might play an important role in the pathogenesis of ischemic cardiomyopathy and could serve as a target in the treatment of this disease (Tur et al., 2013). In addition, LCN2 is identified to mediate neuroinflammation after ischemic and hemorrhagic strokes (Chou, Wang, Kumar, & Weng, 2015). Jin et al. confirmed that LCN2 might contribute to neuronal cell death in the ischemic brain via increasing glial neurotoxicity and promoting neuroinflammation (Jin et al., 2014). Wang et al. also suggested that LCN2 as a neurotoxic factor was rapidly released in response to cerebral ischemia to reduce stroke‐reperfusion injury (Wang et al., 2015). Although the roles of IGFBP‐6 and LYVE1 have not been discussed in our study, we speculate the above cytokines may be key factors involved in MMI progression.

On the other hand, the concentrations of TGFB1, MMP10, IL27, EGF, PDGFA, and CCL2 were significantly lower in patients with MMI compared with those in patients with NACI. Moreover, PDGFA, EGF, CCL2, and TGFB1 were significantly enriched in the pathway of cytokine–cytokine receptor interaction (Figure 1). PDGFA and EGF belong to PDGF family. PDGFA is observed to be involved in atherosclerotic stroke previously (Xu et al., 2008). PDGF signaling is also reported to regulate blood–brain barrier permeability and integrity during ischemic stroke (Su et al., 2008). EGF has been shown to be implicated in neurogenesis and neuroprotection. Intracerebroventricular injection of EGF can enhance nestin expression and reduce neurological deficit and infarct volume after focal cerebral infarction in adult hypertensive rats (Yu et al., 2009), and promoting endogenous neurogenesis by EGF may be a novel therapeutic strategy against ischemic stroke (Teramoto, Qiu, Plumier, & Moskowitz, 2003). In addition, TGFB1 belongs to TGF‐β family which plays an important role in inflammation in stroke and focal cerebral ischemia (Huang, Upadhyay, & Tamargo, 2006). TGF‐β signaling is characterized as a neurotrophic pathway that has protective roles in several central nervous system disorders, including ischemic stroke (Vivien & Ali, 2006). Significantly increased expression levels of PDGFA, EGF, and TGFB1 are observed after preconditioning, which is a phenomenon to induce ischemic tolerance (Naylor, Bowen, Sailor, Dempsey, & Vemuganti, 2005). Besides, CCL2 is one of the chemokines which can interact with CCR2, and CCL2/CCR2 interaction is critical for mediating transendothelial recruitment of intravascularly delivered neural stem cells in response to ischemic brain (Andres et al., 2011). Cerebral CCL2 has also been reported to be responsible for the migration of peripheral immune cell into the brain in neuroinflammatory conditions (Cazareth, Guyon, Heurteaux, Chabry, & Petit‐Paitel, 2014). CCL2 expression is also associated with the infarct volume which plays a pivotal role in brain inflammation and infarct volume development after ischemic stroke (Lee et al., 2015). Although cytokine–cytokine receptor interaction pathway has not been fully discussed in this study, considering the important roles of these cytokines, our results imply that the above down‐regulated cytokines may participate in the progression of MMI via involving in cytokine–cytokine receptor interaction pathway.

Utilizing microarray analysis to detect key cytokines associated with MMI quickly and simultaneously is an obvious advantage of our study. This method will provide a powerful tool for exploring drug targets and diagnostic biomarkers. However, small sample size was the limitation of our study. Moreover, due to lack of clinical samples of MMI and NACI, these key cytokines that we obtained were not confirmed by different molecular assays, such as ELISA, western blot analysis, or other *in vivo* and *in vitro* experiments. Besides, we only compared MMI patients with NACI patients, and not with patients suffering from an acute (nonmalignant) ischemic stroke. A comparison of MMI patients with patients suffering from an acute (nonmalignant) ischemic stroke would clarify much better the significance of the measured cytokines regarding the diagnostic relevance of MMI. Further studies with larger sample sizes and experiments are warranted to validate our microarray results.

In conclusion, we quantified the levels of 320 cytokines in peripheral blood of patients with MMI and patients with NACI, and 10 differentially expressed cytokines, such as NCAM1, LCN2, IGFBP‐6, LYVE1, MMP‐10, IL‐27, PDGFA, EGF, CCL2 and TGFB1 might contribute to the development of MMI. Moreover, cytokine–cytokine receptor interaction pathway may be an important mechanism involved in this disease. These differentially expressed cytokines may serve as diagnostic biomarkers or drug targets for this disease.

## CONFLICT OF INTEREST

None.

## AUTHOR CONTRIBUTIONS

All authors had full access to all the data in the study and take responsibility for the integrity of the data and the accuracy of the data analysis. Study concept and design: Yaling Han, Huisheng Chen, Zhonghe Zhou. Acquisition of data: Zhonghe Zhou, Jinghua Zhang, Xiaoqiu Li. Analysis and interpretation of data: Cheng Xia, Yaling Han. Drafting of the manuscript: Huisheng Chen. Critical revision of the manuscript for important intellectual content: Yaling Han.

## SIGNIFICANCE STATEMENT

We quantified the levels of 320 cytokines in peripheral blood of patients with MMI and patients with NACI, and 10 differentially expressed cytokines, such as NCAM1, LCN2, IGFBP‐6, LYVE1, MMP‐10, IL‐27, PDGFA, EGF, CCL2, and TGFB1 might contribute to the development of MMI. Moreover, cytokine–cytokine receptor interaction pathway may be an important mechanism involved in this disease.

## References

[brb3746-bib-0001] Andres, R. H. , Choi, R. , Pendharkar, A. V. , Gaeta, X. , Wang, N. , Nathan, J. K. , … Steinberg, G. K. (2011). The CCR2/CCL2 interaction mediates the transendothelial recruitment of intravascularly delivered neural stem cells to the ischemic brain. Stroke, 42(10), 2923–2931. 21836091 10.1161/STROKEAHA.110.606368 PMC3371396

[brb3746-bib-0002] Ashburner, M. , Ball, C. A. , Blake, J. A. , Botstein, D. , Butler, H. , Cherry, J. M. , … Eppig, J. T. (2000). Gene Ontology: Tool for the unification of biology. Nature Genetics, 25(1), 25–29. 10802651 10.1038/75556 PMC3037419

[brb3746-bib-0003] Berrouschot, J. , Sterker, M. , Bettin, S. , K02ster, J. , & Schneider, D. (1998). Mortality of space‐occupying (‘malignant’) middle cerebral artery infarction under conservative intensive care. Intensive Care Medicine, 24(6), 620–623. 9681786 10.1007/s001340050625

[brb3746-bib-0004] Cazareth, J. , Guyon, A. , Heurteaux, C. , Chabry, J. , & Petit‐Paitel, A. (2014). Molecular and cellular neuroinflammatory status of mouse brain after systemic lipopolysaccharide challenge: Importance of CCR2/CCL2 signaling. Journal of Neuroinflammation, 11(132), 1742–2094. 10.1186/1742-2094-11-132 PMC4237883 25065370

[brb3746-bib-0005] Chou, W. , Wang, G. , Kumar, V. , & Weng, Y. (2015). Lipocalin‐2 in Stroke. Neuro Open Journal, 2(1), 38–41. 10.17140/NOJ-2-109 PMC6287915 30542675

[brb3746-bib-0006] Chu, H. X. , Kim, H. A. , Lee, S. , Moore, J. P. , Chan, C. T. , Vinh, A. , … Drummond, G. R. (2014). Immune cell infiltration in malignant middle cerebral artery infarction: Comparison with transient cerebral ischemia. Journal of Cerebral Blood Flow & Metabolism, 34(3), 450–459. 24326388 10.1038/jcbfm.2013.217 PMC3948121

[brb3746-bib-0007] Croft, D. , Mundo, A. F. , Haw, R. , Milacic, M. , Weiser, J. , Wu, G. , … Kamdar, M. R. (2014). The Reactome pathway knowledgebase. Nucleic Acids Research, 42(D1), D472–D477. 24243840 10.1093/nar/gkt1102 PMC3965010

[brb3746-bib-0008] Dirnagl, U. , Simon, R. P. , & Hallenbeck, J. M. (2003). Ischemic tolerance and endogenous neuroprotection. Trends in Neurosciences, 26(5), 248–254. 12744841 10.1016/S0166-2236(03)00071-7

[brb3746-bib-0009] Dennis, G. , Sherman, B. T. , Hosack, D. A. , Yang, J. , Gao, W. , Lane, H. C. , & Lempicki, R. A. (2003). DAVID: Database for annotation, visualization, and integrated discovery. Genome Biology, 4(9), R60. PMC193660 12734009

[brb3746-bib-0010] Feuerstein, G. Z. , Wang, X. , & Barone, F. C. (1998). The role of cytokines in the neuropathology of stroke and neurotrauma. NeuroImmunoModulation, 5(3–4), 143–159. 9730680 10.1159/000026331

[brb3746-bib-0011] Frizzell, J. P. (2005). Acute stroke: Pathophysiology, diagnosis, and treatment. AACN Advanced Critical Care, 16(4), 421–440. 10.1097/00044067-200510000-00002 16269890

[brb3746-bib-0012] Hacke, W. , Schwab, S. , Horn, M. , Spranger, M. , & De GM, R. V. K. (1996). Malignant' middle cerebral artery territory infarction: Clinical course and prognostic signs. Archives of Neurology, 53(4), 309–315. 8929152 10.1001/archneur.1996.00550040037012

[brb3746-bib-0013] Hankey, G. J. (2006). Potential new risk factors for ischemic stroke what is their potential? Stroke, 37(8), 2181–2188. 16809576 10.1161/01.STR.0000229883.72010.e4

[brb3746-bib-0014] Huang, J. , Upadhyay, U. M. , & Tamargo, R. J. (2006). Inflammation in stroke and focal cerebral ischemia. Surgical Neurology, 66(3), 232–245. 16935624 10.1016/j.surneu.2005.12.028

[brb3746-bib-0015] Huttner, H. B. , & Schwab, S. (2009). Malignant middle cerebral artery infarction: Clinical characteristics, treatment strategies, and future perspectives. The Lancet Neurology, 8(10), 949–958. 19747656 10.1016/S1474-4422(09)70224-8

[brb3746-bib-0016] Jeong, H. , Mason, S. P. , Barabási, A.‐L. , & Oltvai, Z. N. (2001). Lethality and centrality in protein networks. Nature, 411(6833), 41–42. 11333967 10.1038/35075138

[brb3746-bib-0017] Jin, M. , Kim, J.‐H. , Jang, E. , Lee, Y. M. , Han, H. S. , Woo, D. K. , … Suk, K. (2014). Lipocalin‐2 deficiency attenuates neuroinflammation and brain injury after transient middle cerebral artery occlusion in mice. Journal of Cerebral Blood Flow & Metabolism, 34(8), 1306–1314. 24780901 10.1038/jcbfm.2014.83 PMC4126090

[brb3746-bib-0018] Kanehisa, M. , & Goto, S. (2000). KEGG: Kyoto encyclopedia of genes and genomes. Nucleic Acids Research, 28(1), 27–30. 10592173 10.1093/nar/28.1.27 PMC102409

[brb3746-bib-0019] Kawada, H. , Takizawa, S. , Takanashi, T. , Morita, Y. , Fujita, J. , Fukuda, K. , … Hotta, T. (2006). Administration of hematopoietic cytokines in the subacute phase after cerebral infarction is effective for functional recovery facilitating proliferation of intrinsic neural stem/progenitor cells and transition of bone marrow‐derived neuronal cells. Circulation, 113(5), 701–710. 16461843 10.1161/CIRCULATIONAHA.105.563668

[brb3746-bib-0020] Kim, H.‐M. , Shin, H.‐Y. , Jeong, H.‐J. , An, H.‐J. , Kim, N.‐S. , Chae, H.‐J. , … Baek, S.‐H. (2000). Reduced IL‐2 but elevated IL‐4, IL‐6, and IgE serum levels in patients with cerebral infarction during the acute stage. Journal of Molecular Neuroscience, 14(3), 191–196. 10984195 10.1385/JMN:14:3:191

[brb3746-bib-0021] Kohl, M. , Wiese, S. , & Warscheid, B. (2011). Cytoscape: Software for visualization and analysis of biological networks. Methods in Molecular Biology, 696, 291–303. 21063955 10.1007/978-1-60761-987-1_18

[brb3746-bib-0022] Lee, S. , Chu, H. X. , Kim, H. A. , Real, N. C. , Sharif, S. , Fleming, S. B. , … Sobey, C. G. (2015). Effect of a broad‐specificity chemokine‐binding protein on brain leukocyte infiltration and infarct development. Stroke, 46(2), 537–544. 25538201 10.1161/STROKEAHA.114.007298

[brb3746-bib-0023] Mergenthaler, P. , Dirnagl, U. , & Meisel, A. (2004). Pathophysiology of stroke: Lessons from animal models. Metabolic Brain Disease, 19(3–4), 151–167. 15554412 10.1023/b:mebr.0000043966.46964.e6

[brb3746-bib-0024] Mi, H. , & Thomas, P. (2009). PANTHER pathway: An ontology‐based pathway database coupled with data analysis tools. Methods in Molecular Biology, 563, 123–140. 19597783 10.1007/978-1-60761-175-2_7 PMC6608593

[brb3746-bib-0025] Minnerup, J. , Wersching, H. , Ringelstein, E. B. , Heindel, W. , Niederstadt, T. , Schilling, M. , … Kemmling, A. (2011). Prediction of malignant middle cerebral artery infarction using computed tomography‐based intracranial volume reserve measurements. Stroke, 42(12), 3403–3409. 21903965 10.1161/STROKEAHA.111.619734

[brb3746-bib-0026] Naylor, M. , Bowen, K. K. , Sailor, K. A. , Dempsey, R. J. , & Vemuganti, R. (2005). Preconditioning‐induced ischemic tolerance stimulates growth factor expression and neurogenesis in adult rat hippocampus. Neurochemistry International, 47(8), 565–572. 16154234 10.1016/j.neuint.2005.07.003

[brb3746-bib-0027] Nishimura, D. (2001). BioCarta. Biotech Software & Internet Report: The Computer Software Journal for Scient, 2(3), 117–120.

[brb3746-bib-0028] Schäbitz, W.‐R. , Kollmar, R. , Schwaninger, M. , Juettler, E. , Bardutzky, J. , Schölzke, M. , … Schwab, S. (2003). Neuroprotective effect of granulocyte colony–stimulating factor after focal cerebral Ischemia. Stroke, 34(3), 745–751. 12624302 10.1161/01.STR.0000057814.70180.17

[brb3746-bib-0029] Schaefer, C. F. , Anthony, K. , Krupa, S. , Buchoff, J. , Day, M. , Hannay, T. , & Buetow, K. H. (2009). PID: The pathway interaction database. Nucleic Acids Research, 37(suppl 1), D674–D679. 18832364 10.1093/nar/gkn653 PMC2686461

[brb3746-bib-0030] Schwab, S. (1998). Early hemicraniectomy in patients with complete middle cerebral artery infarction. Stroke, 29(9), 1888–1893. 9731614 10.1161/01.str.29.9.1888

[brb3746-bib-0031] Sotgiu, S. , Zanda, B. , Marchetti, B. , Fois, M. L. , Arru, G. , Pes, G. M. , … Rosati, G. (2006). Inflammatory biomarkers in blood of patients with acute brain ischemia. European Journal of Neurology, 13(5), 505–513. 16722977 10.1111/j.1468-1331.2006.01280.x

[brb3746-bib-0032] Sparacia, G. , Iaia, A. , Assadi, B. , & Lagalla, R. (2007). Perfusion CT in acute stroke: Predictive value of perfusion parameters in assessing tissue viability versus infarction. La Radiologia Medica, 112(1), 113–122. 17310286 10.1007/s11547-007-0125-9

[brb3746-bib-0033] Su, E. J. , Fredriksson, L. , Geyer, M. , Folestad, E. , Cale, J. , Andrae, J. , … Yepes, M. (2008). Activation of PDGF‐CC by tissue plasminogen activator impairs blood‐brain barrier integrity during ischemic stroke. Nature Medicine, 14(7), 731–737. 10.1038/nm1787 PMC2811427 18568034

[brb3746-bib-0034] Szklarczyk, D. , Franceschini, A. , Kuhn, M. , Simonovic, M. , Roth, A. , Minguez, P. , … Bork, P. (2011). The STRING database in 2011: Functional interaction networks of proteins, globally integrated and scored. Nucleic Acids Research, 39(suppl 1), D561–D568. 21045058 10.1093/nar/gkq973 PMC3013807

[brb3746-bib-0035] Teramoto, T. , Qiu, J. , Plumier, J. C. , & Moskowitz, M. A. (2003). EGF amplifies the replacement of parvalbumin‐expressing striatal interneurons after ischemia. Journal of Clinical Investigation, 111(8), 1125–1132. 12697732 10.1172/JCI17170 PMC152938

[brb3746-bib-0036] Tur, M. K. , Etschmann, B. , Benz, A. , Leich, E. , Waller, C. , Schuh, K. , … Haaf, A. T. (2013). The 140‐kD Isoform of CD56 (NCAM1) Directs the Molecular Pathogenesis of Ischemic Cardiomyopathy. American Journal of Pathology, 182(4), 1205–1218. 23462508 10.1016/j.ajpath.2012.12.027

[brb3746-bib-0037] Tuttolomondo, A. , Di Raimondo, D. , di Sciacca, R. , Pinto, A. , & Licata, G. (2008). Inflammatory cytokines in acute ischemic stroke. Current Pharmaceutical Design, 14(33), 3574–3589. 19075734 10.2174/138161208786848739

[brb3746-bib-0038] Vahedi, K. , Hofmeijer, J. , Juettler, E. , Vicaut, E. , George, B. , Algra, A. , … Rothwell, P. M. (2007). Early decompressive surgery in malignant infarction of the middle cerebral artery: A pooled analysis of three randomised controlled trials. The Lancet Neurology, 6(3), 215–222. 17303527 10.1016/S1474-4422(07)70036-4

[brb3746-bib-0039] Vila, N. , Castillo, J. , Dávalos, A. , & Chamorro, Á. (2000). Proinflammatory cytokines and early neurological worsening in ischemic stroke. Stroke, 31(10), 2325–2329. 11022058 10.1161/01.str.31.10.2325

[brb3746-bib-0040] Vivien, D. , & Ali, C. (2006). Transforming growth factor‐β signalling in brain disorders. Cytokine Growth Factor Reviews, 17(1), 121–128. 16271500 10.1016/j.cytogfr.2005.09.011

[brb3746-bib-0041] Wang, G. , Weng, Y. C. , Han, X. , Whaley, J. D. , McCrae, K. R. , & Chou, W. H. (2015). Lipocalin‐2 released in response to cerebral ischaemia mediates reperfusion injury in mice. Journal of Cellular and Molecular Medicine, 19(7), 1637–1645. 25702801 10.1111/jcmm.12538 PMC4511361

[brb3746-bib-0042] Wang, J. , Zhou, D. , Qin, H. , Xu, Y. , Guan, Y. , & Zang, W. (2013). Screening of key genes associated with ischemic stroke via microarray data. Canadian Journal of Neurological Sciences, 40(06), 864–869. 24257231 10.1017/s0317167100016036

[brb3746-bib-0043] Wilson, D. S. , & Nock, S. (2003). Recent developments in protein microarray technology. Angewandte Chemie International Edition, 42(5), 494–500. 12569479 10.1002/anie.200390150

[brb3746-bib-0044] Xu, H. , Tang, Y. , Liu, D.‐Z. , Ran, R. , Ander, B. P. , Apperson, M. , … Pancioli, A. (2008). Gene expression in peripheral blood differs after cardioembolic compared with large‐vessel atherosclerotic stroke: Biomarkers for the etiology of ischemic stroke. Journal of Cerebral Blood Flow & Metabolism, 28(7), 1320–1328. 18382470 10.1038/jcbfm.2008.22

[brb3746-bib-0045] Yu, J. , Zeng, J. , Cheung, R. T. , Xiong, L. , He, M. , Liang, Z. , … Huang, R. (2009). Intracerebroventricular injection of epidermal growth factor reduces neurological deficit and infarct volume and enhances nestin expression following focal cerebral infarction in adult hypertensive rats. Clinical and Experimental Pharmacology and Physiology, 36(5–6), 539–546. 19673937 10.1111/j.1440-1681.2008.05105.x

